# Use of machine learning to identify functional connectivity changes in a clinical cohort of patients at risk for dementia

**DOI:** 10.3389/fnagi.2022.962319

**Published:** 2022-09-01

**Authors:** Ying Shen, Qian Lu, Tianjiao Zhang, Hailang Yan, Negar Mansouri, Karol Osipowicz, Onur Tanglay, Isabella Young, Stephane Doyen, Xi Lu, Xia Zhang, Michael E. Sughrue, Tong Wang

**Affiliations:** ^1^Rehabilitation Medicine Center, The First Affiliated Hospital of Nanjing Medical University, Nanjing, China; ^2^Department of Rehabilitation Medicine, The Affiliated Jiangsu Shengze Hospital of Nanjing Medical University, Suzhou, China; ^3^Department of Radiology, The Affiliated Jiangsu Shengze Hospital of Nanjing Medical University, Suzhou, China; ^4^Omniscient Neurotechnology, Sydney, NSW, Australia; ^5^Department of Rehabilitation Medicine, China-Japan Friendship Hospital, Beijing, China; ^6^International Joint Research Center on Precision Brain Medicine, XD Group Hospital, Xi’an, China; ^7^Shenzhen Xijia Medical Technology Company, Shenzhen, China

**Keywords:** dementia, functional connectivity, Alzheimer’s disease, graph theory, brain network

## Abstract

**Objective:**

Progressive conditions characterized by cognitive decline, including mild cognitive impairment (MCI) and subjective cognitive decline (SCD) are clinical conditions representing a major risk factor to develop dementia, however, the diagnosis of these pre-dementia conditions remains a challenge given the heterogeneity in clinical trajectories. Earlier diagnosis requires data-driven approaches for improved and targeted treatment modalities.

**Methods:**

Neuropsychological tests, baseline anatomical T1 magnetic resonance imaging (MRI), resting-state functional MRI (rsfMRI), and diffusion weighted scans were obtained from 35 patients with SCD, 19 with MCI, and 36 age-matched healthy controls (HC). A recently developed machine learning technique, Hollow Tree Super (HoTS) was utilized to classify subjects into diagnostic categories based on their FC, and derive network and parcel-based FC features contributing to each model. The same approach was used to identify features associated with performance in a range of neuropsychological tests. We concluded our analysis by looking at changes in PageRank centrality (a measure of node hubness) between the diagnostic groups.

**Results:**

Subjects were classified into diagnostic categories with a high area under the receiver operating characteristic curve (AUC-ROC), ranging from 0.73 to 0.84. The language networks were most notably associated with classification. Several central networks and sensory brain regions were predictors of poor performance in neuropsychological tests, suggesting maladaptive compensation. PageRank analysis highlighted that basal and limbic deep brain region, along with the frontal operculum demonstrated a reduction in centrality in both SCD and MCI patients compared to controls.

**Conclusion:**

Our methods highlight the potential to explore the underlying neural networks contributing to the cognitive changes and neuroplastic responses in prodromal dementia.

## Introduction

Alzheimer’s disease (AD) is the most common form of dementia, characterized by progressive neurodegeneration with resulting memory decline and cognitive impairment that interferes with activities of independent living (Ballard et al., 2011; McKhann et al., 2011). Mild cognitive impairment (MCI) is a prodromal stage of impairment where patients have a higher risk of progressing to AD (Vega and Newhouse, 2014), with an estimated progression at a rate of approximately 10–15% per year (Michaud et al., 2017). Additionally, patients with MCI may remain stable, or rarely return to a cognitively unimpaired state (Giorgio et al., 2020). Recently, the earliest stage of noticeable symptoms of dementia, subjective cognitive decline (SCD), has been explored as a prodromal risk factor for AD, providing additional insights and obfuscation in disease trajectory. AD neuropathology, which includes aggregation and deposition of amyloid- β peptide (Aβ) and tau protein, also begins around 10–20 years prior to the onset of symptoms (Villemagne et al., 2013), further complicating the uncertainty in individual patient trajectory. Given this uncertainty, it is necessary to examine the neurological basis of higher cognitive functioning differentiating pre-dementia status (SCD and MCI) from normal aging, in order to develop accurate and clinically reliable tools identifying individuals who may develop MCI and AD early in the disease course. Reliable biomarkers and predictors of AD trajectory may enable therapeutic interventions to delay progression or even modify disease at a pre-dementia stage.

While the utility of several imaging and fluid biomarkers in AD diagnosis have been investigated, resting state functional magnetic resonance imaging (rsfMRI) remains a popular choice due to its non-invasive nature and spatial resolution. rsfMRI enables the examination of changes in functional connectivity (FC) across large-scale brain networks, providing information on early changes in the disease course, which have been identified up to 4 years prior to the symptomatic onset of AD (Chiesa et al., 2019; Wisch et al., 2020; Song et al., 2022; Wang et al., 2022). Previously, Shi and Liu extracted features from rsfMRI to classify several stages of MCI with high accuracy (Shi and Liu, 2020); Wee et al. (2012) utilized support vector machines on multimodal data combining functional and structural connectivity to further improve the accuracy of MCI classification; and Zhu et al. (2022) classified AD, MCI and healthy controls (HC) using hippocampus seed based FC. So far, these machine learning techniques have mainly been used for diagnostic classification of rsfMRI based on predefined regions of interest with known FC changes. This is fundamentally due to the difficulty in attaining feature importance in machine learning models with a large number of features, as in the whole brain (Doyen et al., 2021b). As a result, the power of machine learning has not been harnessed to directly study and identify the underlying whole network-based FC changes contributing to the model’s classification of diagnosis. This is crucial given that AD is defined as a disconnection syndrome across multiple networks (Seeley et al., 2009; Wang et al., 2013). Additionally, there has been little focus on examining these changes in the earliest stages of pathology, particularly SCD, the earliest stage of noticeable symptoms of dementia.

In this study, we applied a recently described machine learning technique to FC-based analysis to build binarized classification models differentiating cohort of health controls, SCD, and MCI. Extracting the feature importance from these models, we performed further analysis to identify anatomical and network-based patterns among the cohorts to examine whether the models could identify similar FC changes demonstrated in the literature from the raw data without biased feature selection. In the second part of our analysis, we applied the same technique to several neuropsychological assessments commonly used in AD in an attempt to identify patterns of neuroanatomical deficit which may be associated with performance in each test. Finally, we applied a graph theory metric, PageRank centrality, in order to examine changes in the importance of brain regions in pre-dementia status compared to healthy controls. We hope that validation of our methods will provide a basis to improve our understanding and diagnosis of MCI and AD.

## Materials and methods

### Patient cohort

The study was conducted in the rehabilitation medicine department of the Jiangsu Shengze Hospital, affiliated with the Nanjing Medical University in the Suzhou Jiangsu Province. The study was approved by the hospital’s Human Research Ethics Committee and registered with the Chinese Clinical Trials Registry (ChiCTR2100046131). In accordance with the Helsinki Declaration, the written consent of the participants was obtained. MCI and SCD participants were from the Department of Rehabilitation Medicine’s memory disorder outpatient clinic. Healthy controls were recruited through advertisements in the hospital, newspapers and through public recruitment methods.

A total of 90 eligible participants were eventually included based on inclusion and exclusion criteria from May to September 2021. This research included three types of elderly subjects (55–80 years old) with varying cognitive levels, including MCI, SCD, and HC. All participants were clinically examined by a neurologist for neuropsychological test batteries as well as a fMRI brain scan prior to participation.

The following were the exclusion criteria for all three groups: (1) a clinical diagnosis of vascular dementia or dementia based on the NINDS-AIREN criteria; (2) Modified Hachinski Ischemic Scores > 4; (3) MMSE scores < 24; (4) drug or alcohol abuse for at least 6 months; (5) could not undergo MRI examinations or neuropsychological tests; (6) Severe cardiovascular or cerebrovascular disease, as well as psychological illness; (7) treatment with cholinesterase inhibitors or NMDA antagonists within 2 weeks before assessments; (8) individuals over the age of 80 and under the age of 55; (9) geriatric depression scale scores ≥ 6; (10) MRI contraindications such as presence of known claustrophobia, ferromagnetic implants, cardiac pacemakers, joint replacement (e.g., hip, knee, etc.), or hearing aids.

Participants were assigned to the MCI group if any one of the three following criteria were met (Bondi et al., 2014; Zhong et al., 2021): (1) had an impaired score (> 1 *SD* below the age-corrected normative mean) on both assessments in at least one cognitive domain (memory, language, or executive function); (2) had one impaired score (> 1 *SD* below the age-corrected normative mean), in each of the three cognitive domains (memory, language, or executive function); or (3) Functional Assessment Questionnaire (FAD) scores ≥ 9. The standard deviation (SD)-based cut-offs utilized in our investigation were based on published studies in the Chinese population that had been adjusted for education and age (Li et al., 2019).

Participants were assigned to the SCD group if they met the inclusion criteria as follows (Jessen et al., 2014, 2020): (1) normal performance on the above-mentioned standard neuropsychological assessment used to diagnose MCI; (2) self-experienced persistent memory decline for at least 6 months, compared to a previous normal condition, which is not related to an acute incident; (3) Self-reported concerns about memory decline. The second and third criteria were derived from a semi-structured clinical interview, and were based on whether the participant expressed persistent memory decline and concerns about this memory decline. The interview involved an initial open-ended question, “Have you noticed any changes in your mental abilities? Could you provide an example?” This was followed by specific questions about each cognitive domain (memory, language, planning, attention, any other cognitive decline).

The following were the inclusion criteria for the HC group: (1) had no self-reported memory issues and no self-reported chronic memory deterioration; (2) MMSE score > 26 and MoCA score > 26; (3) clinical dementia rating score = 0.

### Neuropsychological testing

The neuropsychological evaluations included global cognitive function and language function. Global cognitive function was evaluated by Montreal Cognitive Assessment Test (MoCA) and Mini-Mental State Examination (MMSE). The language function was measured by the Boston Naming Test China version (BNT-C) and Animal Fluency test (AFT). Learning was assessed using the Rey Auditory Verbal Learning Test (AVLT).

### Statistical analysis

Group differences in the continuous variables were analyzed using a non-parametric Kruskal-Wallis one-way analysis of variance. If an instance was rejected, *post hoc* Dunn’s test with Bonferroni correction for multiple comparisons was performed for pairwise comparisons of a given variable between each of the three diagnostic groups. The only categorical variable, sex, was compared between groups using a Chi-squared test of independence. All statistical analysis was performed in R version 4.1.0.

### Imaging protocol

All subjects underwent an MRI scan on a 3.0T GE Discovery MR750w (SIGNA) scanner 24 channel head coil (Head 24) 10 min A gradient-echo echo-planar imaging T2* sensitive pulse sequence was used to acquire resting-state fMRI data (interleaved sequence, slices = 41, thickness = 3.5 mm, pixel spacing = 3 × 3 mm, repetition time (TR) = 2,500 ms, echo time (TE) = 30 ms, field of view (FOV) = 192 × 192 mm, flip angle = 90°, and acquisition matrix = 64 × 64, percent sampling = 100%, percent Phase Field of View = 100%).

A three-dimensional 5 min spoiled-gradient recalled T1-weighted sequence (axial 3D T1 BRAVO) was used to acquire whole-brain structural data with an acquisition time of 301 s (slices = 188, thickness = 1 mm, pixel spacing = 1*1 mm, TR = 8.5 ms, TE = 3.2 ms, Inversion Time = 450 ms, Spacing Between Slice = 1, skip = 0 mm, flip angle = 12°, FOV = 256 × 256 mm, and acquisition matrix = 256 × 256, percent sampling = 100%, percent Phase Field of View = 100%).

Diffusion-weighted volumes parameters were used: 65 contiguous slices, slice thickness = 2.1 mm, FOV = 256 × 256 mm, matrix = 128 × 128 mm, TR = 17,000 ms, TE = 95.9 ms, voxel size = 2 mm isotropic, acquisition NEX = 1 partial Fourier, 64 diffusion directions with *b*-value = 1,000 s/mm^2^, and 1 image with no diffusion weighting (b = 0 s/mm^2^), bandwidth = 250 Hz/pixel. Acquisition time was 19.16 min per DTI scan.

### Diffusion weighted imaging preprocessing

The diffusion weighted imaging (DWI) images were processed using the Infinitome software (Omniscient Neurotechnology, 2020), which employs standard processing steps in the Python language. The processing pipeline includes the following: (1) the diffusion image is resliced to ensure isotropic voxels, (2) motion correction is performed using a rigid body registration algorithm to a baseline scan, (3) slices with excess movement (defined as DVARS > 2 sigma from the mean slice) are eliminated, (4) the T1 image is skull stripped using the HD-BET software (Isensee et al., 2019), which is inverted and aligned to the DWI image using a rigid alignment, and used as a mask to skull strip the aligned DWI image, (5) gradient distortion correction is performed by applying a diffeomorphic warping registration method between the DWI and T1 images, (6) The fiber response function is estimated and the diffusion tensors are calculated using constrained spherical deconvolution, (7) deterministic tractography is performed with uniform random seeding, 4 seeds per voxel, usually creating about 300,000 streamlines per brain.

### Structural connectivity based parcellation

Identifying FC changes at a granular anatomical level requires the application of a parcellation scheme to the brain. While several atlases are available to define brain regions, many methods rely on healthy cortices for parcellation. Furthermore, most of the available atlases parcellate a given scan based on the group average of these healthy cohorts, risking inaccurate parcellation due to gyral variation or morphological differences brought on by pathology. In order to minimize this, we adopted a machine-learning based method to create subject-specific versions of the Human Connectome Project Multimodal Parcellation (HCP-MMP1) atlas (Glasser et al., 2016). While this method is described in detail elsewhere (Doyen et al., 2021a), briefly, a machine learning model was trained using tractography data from 178 healthy controls obtained from the SchizConnect database, preprocessed as above, in order for it to learn the structural connectivity pattern between voxels included within the 379 parcels of the HCP-MMP1 atlas. The same unaltered atlas was then warped onto each brain in the study sample and the trained machine learning model was applied to each subject to re-appoint voxels located at the endpoint of tractography streamlines to their most likely warped parcellation based on the structural connectivity feature vectors. This resulted in reparcellation of voxels, creating a version of the HCP-MMP1 atlas with 180 cortical parcels and 9 subcortical structures per hemisphere, along with the brainstem as one parcel.

The network affiliation of each HCP parcel was based on the automatic mapping provided by the Infinitome software, which itself is based on previous meta-analyses exploring each large-scale network. The networks included in this template were the core networks described by Yeo et al. (2011), Central Executive Network (CEN), Default Mode Network (DMN), Dorsal Attention Network (DAN), Limbic Network (LN), Salience Network (SN), Sensorimotor Network (SMN), and the Visual Network (VN), along with several networks which are either part of the extended versions of the core networks, or additional networks described in the literature, including the Accessory Language and Language Networks (part of the extended DMN), Auditory System (part of the SMN), Multiple demand network, and the Ventral Attention Network (VAN).

### rsfMRI preprocessing steps

The rsfMRI images were processed using standard processing steps including: (1) motion correction on the T1 and BOLD images using a rigid body alignment, (2) elimination of slices with excess movement (defined as DVARS > 2 sigma from the mean slice), (3) skull stripping of the T1 image using a convolutional neural net (CNN), which is inverted and aligned to the resting state bold image using a rigid alignment, and used as a mask to skull strip the rsfMRI image, (4) slice timing correction, (5) Global intensity normalization, (6) gradient distortion correction using a diffeomorphic warping method to register the rsfMRI and T1 images, (7) High variance confounds are calculated using the CompCor method (Behzadi et al., 2007); these confounds as well as motion confounds are regressed out of the rsfMRI image, and the linear and quadratic signals are detrended. Note this method does not perform global signal regression, (8) spatial smoothing is performed using a 4 mm FWHM Gaussian kernel. The personalized atlas created in previous steps is registered to the T1 image, and gray matter atlas regions are aligned with the gray matter regions in each subject’s scans. Thus, it is ideally positioned for extracting a BOLD time series, averaged over all voxels within a region, from all 379 regions (180 parcels from two hemispheres, plus 19 subcortical structures). The Pearson correlation coefficient is calculated between the BOLD signals of each unique area pair (self to self-inclusive), which yields 143,641 correlations.

### Machine learning classification and feature extraction using the hollow-tree super method

Machine learning was used to model the diagnostic group and neuropsychological test performance of each participant based on the pairwise functional correlation between the 379 regions of each individual’s brain atlas. In each instance, XGB Classifier, a boosted trees approach was used to fit the model. This approach provides a superior prediction ability than single trees. All models included age and sex as nuisance predictors. For the diagnostic group modeling, four models were trained to classify SCD from HC, MCI from HC, a combined SCD and MCI cohort (herein referred to as pre-dementia status) from HC, and SCD from MCI. For the test performance models, the four models trained were for the MMSE, MoCA, BNT, and AFT.

The black box problem in machine learning generally limits the ability to utilize machine learning techniques in clinical practice, as there is a need-to-know which parts of the brain are associated with pathology. However, extracting features from machine learning models is limited when working with large datasets, especially when investigating the magnitude and directionality of features on classification. In order to circumvent this, we used a technique described recently elsewhere, Hollow Tree Super (HoTS) (Doyen et al., 2021b). HoTS linearizes decision trees in order to provide directional feature importance coefficients. Consequently, for each model, we can obtain a list of FC features, corresponding to pairwise HCP parcels, along with an indication of their impact on the overall model. We used fivefold cross-validation, and evaluated each model with the mean area under the receiver operating characteristic curve (AUC-ROC) ± standard deviation. In order to mitigate the effects of class imbalances in the models, we employ three methodological approaches: (1) we do stratified fivefold cross validation to make sure each fold has an equal ratio of both classes, (2) we implement a stopping criteria, whereby we stop training if performance doesn’t improve over consecutive iterations of hyper-parameter tuning, to prevent overfitting (e.g., to the larger class for example). These guardrails therefore ensures that our modeling approach therefore approximates a balanced class AUC even in instances of class imbalance. Finally, our feature importance calculation only draws inferences on the correctly predicted cases; so it matters less than one class might be predicted slightly better than the other. In this way, our predominant aim is isolating what drives the classifications. Therefore, while AUC is inherently sensitive to class imbalances, we can assume that our corrections and significantly higher than chance performance make nuances in AUC bias irrelevant to the purpose of our analysis.

In some instances where the model achieved a lower AUC, we used random forest-based feature reduction to threshold a higher AUC. It is shown that a random forest algorithm is suitable when there are more features than observations, as in the current study, and is based on an embedded feature selection which incorporates interactions between features. For each model, we produced a representation of the importance of each network in the output of the model, and a SHAP plot of the top 20 features contributing to the model. The SHAP method calculates feature importance by deriving Shapley values for each feature, a technique derived from cooperative game theory. Shapley values consider feature importance based on estimating the marginal contribution of each feature to the outcome of models with every possible permutation of features (Lundberg and Lee, 2017). Each SHAP plot provides a list of features in descending order of importance, along with their impact on the model along the x-axis, with the color of each point. representing a single observation, indicating whether a high (red), or low (blue) value of that feature is associated with the model.

For the models classifying subjects into neuropsychological test performance based on their FC, each test outcome was binarized into poor and good performance. In order to reflect their clinical use, all neuropsychological scores were binarized according to accepted cutoff scores. For the MMSE, we used an aggressive cutoff of ≤ 28, aligning with a recent study demonstrating a high sensitivity of this cutoff in MCI (De Marchis et al., 2010); for the MoCA, a score greater than 25 was considered normal (Milani et al., 2018); for the BNT, a score greater than 23 was considered normal (Li et al., 2022); and for the AFT, a score ≤ 15 was considered abnormal (Canning et al., 2004).

## Results

### Subject characteristics

Subject demographics are presented in Table 1. There was no significant difference in sex distributions, age, or education between groups. There was however, a significant difference in the MMSE [H (2) = 31.10, *p* < 0.001], MoCA [H (2) = 48.83, *p* < 0.001], BNT [H (2) = 22.08, *p* < 0.001], and AFT [H (2) = 13.47, *p* = 0.001] scores between groups. *Post hoc* Dunn’s test was used to compare all pairs of groups. The MMSE was significantly different between MCI and HC (*p* < 0.001), and SCD and MCI (*p* = 0.002). The MoCA showed a significant difference between all groups (*p* < 0.001). The BNT was significantly different between MCI and HC (*p* < 0.001), and SCD and MCI (*p* = 0.002). Finally, the AFT was only significantly different between MCI and HC (*p* < 0.001).

**TABLE 1:** Subject demographics.

Variable	Healthy controls (*n* = 36)	Subject cognitive decline (*n* = 35)	Mild cognitive impairment (*n* = 19)	*P*-value
Sex F/M (%)	25/11 (69.4/30.6)	24/11 (68.6/31.4)	16/3 (84.2/15.8)	0.421
Median age (IQR) years	67.0 (7.3)	64.0 (8.5)	70.0 (6.0)	0.086
Median years of education (IQR)	9.0 (3.0)	9.0 (3.0)	9.0 (3.0)	0.935
Median MMSE score (IQR)	30 (0)	29 (2)	28 (2)	< 0.001
Median MoCA score (IQR)	28 (2.0)	26 (3.0)	22 (4.5)	< 0.001
Median BNT score (IQR)	24.5 (4.0)	24.0 (4.0)	20.0 (4.5)	< 0.001
Median AFT score (IQR)	16 (4.5)	15 (5.0)	13 (2.0)	0.001

MMSE, Mini Mental State Exam; MoCA, Montreal Cognitive Assessment; BNT, Boston Naming Test; AFT, Animal Fluency Test.

### Functional connectivity changes in language networks may arise early in Alzheimer’s disease

**FIGURE 1:**
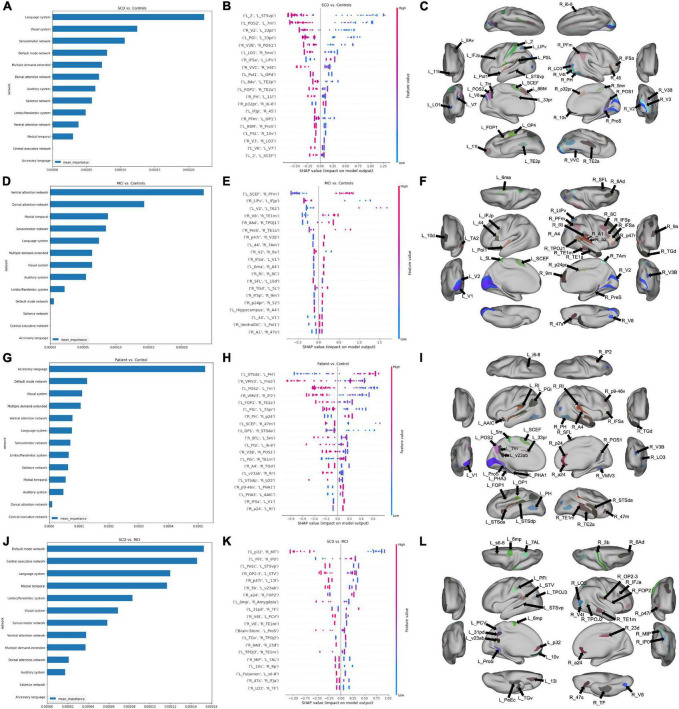
Features associated with each machine learning model classifying subjects into diagnostic groups. **(A)** A graph ranking network level features, and **(B)** a SHAP plot ranking the top 20 parcel-based features when classifying SCD from controls, along with, **(C)** an anatomical representation of the parcels comprising the top 20 features. **(D)** Network level graph, **(E)** SHAP plot, and **(F)** anatomical representation for the model classifying MCI and controls. **(G)** Network level graph, **(H)** SHAP plot, and **(I)** anatomical representation for the model classifying the combined SCD and MCI cohort, and controls. **(J)** Network level graph, **(K)** SHAP plot, and **(L)** anatomical representation for the model classifying SCD and MCI.

Our model classifying cases into either SCD or HC attained a mean test AUC of 0.74 ± 0.095. At the network level, the language network was the strongest predictor of group membership (Figure 1A). At the parcel level, a low level of the correlation between area left 2 and area left STSvp, area left POS2 and left 7 m, area right V2 and left 33 pr had a positive impact on the group prediction (Figure 1B). There was, however, a high level of variance in parcel-wise connections, making it difficult to identify a single most influential connection. Anatomically, out of the top 20 features in the model, most of the parcels were in the perisylvian and occipital regions (Figure 1C).

Next, the classification of MCI and HC yielded a mean test AUC of 0.77 ± 0.11. The mean AUC increased to 0.84 following feature reduction to 350 features. The VAN, followed by the DAN were the top two strongest predictors of group differences (Figure 1D). A higher correlation between the left SCEF and right PFm had a high positive impact on group prediction (Figure 1E). Out of the top 20 features, a majority of parcels were located in the right prefrontal and temporal regions. The left primary (left V1), and secondary visual areas bilaterally (V2), were also included, among other occipital regions (Figure 1F).

Classification of SCD + MCI (combined pre-dementia) from HC attained a mean test AUC of 0.73 ± 0.11. Reducing the number of features using random forest feature selection increased mean AUC to 0.82 with 678 features. Regions in the accessory language network were the best predictors of SCD + MCI and HC classification (Figure 1G). A high positive correlation of the left STSda and left PH had a positive impact on group prediction, while a negative correlation between right VMV3 and left ProS was associated with prediction (Figure 1H). Among the top 20 features, a majority of parcels were in the temporal lobe bilaterally (Figure 1I).

Finally, classification of SCD and MCI attained a mean test AUC of 0.84 ± 0.13. Feature reduction worsened model performance, lowering mean test AUC to 0.77 ± 0.12. Both the DMN and CEN demonstrated differences in connectivity between SCD and MCI patients (Figure 1J). A low level of correlation between the left p32 and right MT was associated with a positive impact on patient group prediction (Figure 1K). The top 20 features were found across several anatomical regions, including the left posterior cingulate and right temporal regions (Figure 1L).

### Patterns in model features may provide diagnostic markers to differentiate disease states

**FIGURE 2:**
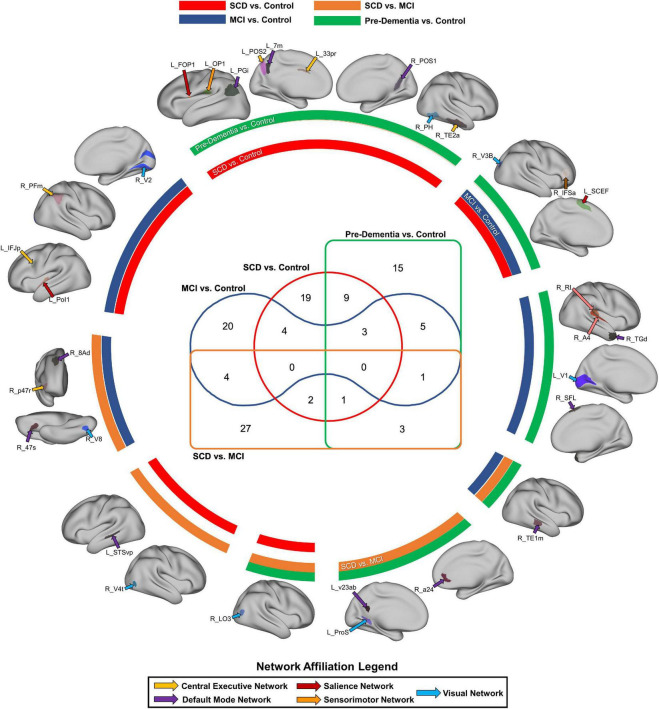
Common parcels associated with at least two classification models. The color of the circular bands represents the associated model, with SCD vs. Control in red, MCI vs. control in blue, pre-dementia status (SCD + MCI) in green, and SCD vs. MCI in orange. The color of the arrows labeling each anatomical region also represents the network affiliated with each region, with a legend provided at the bottom of the figure. The Venn diagram in the middle signifies the number of parcels which are common to at least two models.

In an attempt to identify potentially important anatomical regions which were included among the top 20 features of each binary classification model, we analyzed the amount of crossover between model features. Figure 2 represents a Venn diagram comparing each model. The greatest amount of crossover among the top 20 features was between regions differentiating SCD and controls, and SCD + MCI and controls. This comprised nine regions, including two insular regions (left area FOP1 and left area OP1), the left angular gyrus (area left PGi), area 7 m in the precuneus, areas of the parietooccipital sulcus bilaterally (left POS2 and right POS1), and two temporooccipital regions (right TE2a and right PH).

### Nociferous compensation by executive networks may be responsible for cognitive deficits in Alzheimer’s disease

**FIGURE 3:**
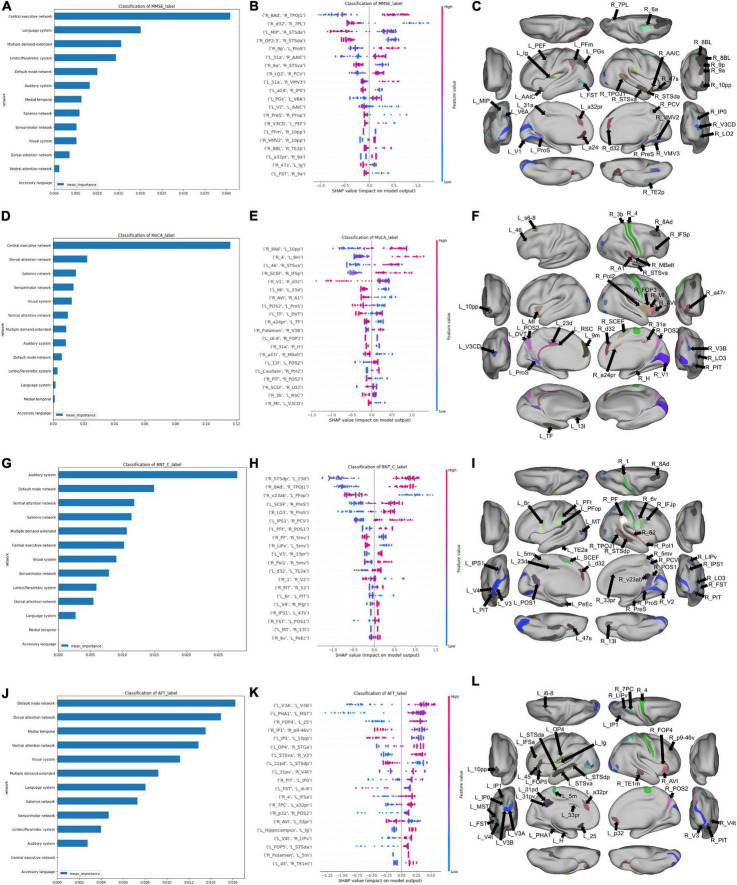
Features associated with each machine learning model classifying subjects into neuropsychological performance. **(A)** A graph ranking network level features, and **(B)** a SHAP plot ranking the top 20 parcel-based features when classifying MMSE performance, along with, **(C)** an anatomical representation of the parcels comprising the top 20 features. **(D)** Network level graph, **(E)** SHAP plot, and **(F)** anatomical representation for the model classifying MoCA performance. **(G)** Network level graph, **(H)** SHAP plot, and **(I)** anatomical representation for the model classifying BNT performance. **(J)** Network level graph, **(K)** SHAP plot, and **(L)** anatomical representation for the model classifying AFT performance.

Overall, the CEN was the strongest predictor of performance in the MMSE and MoCA, though both of these network analyses were based on a reduced number of features, and thus must be interpreted cautiously (Figures 3A,D). The MMSE model with the entire connectome as its input achieved a mean AUC of 0.53 ± 0.16, which rose to 0.74 ± 0.07 after feature reduction to 698 features. Following feature reduction, a positive correlation between the right 8 Ad and the right temporoparietooccipital junction 1 (right TPOJ1), both DMN parcels, and a negative correlation between the right d32, a CEN parcel, and right lateral area 7P (right 7PL), a DAN parcel, were most associated with the model’s classification (Figure 3B). Anatomically, the left parietooccipital and right frontotemporal regions were overrepresented among the top 20 features of the model (Figure 3C).

The model predicting MoCA performance initially achieved a mean AUC of 0.45 ± 0.14, and 0.78 ± 0.08 following feature reduction. The latter model demonstrated that a high correlation between the right 8 Ad and left 10 pp, right area 4 and left 9 m, left area 46 and right STSva, and right SCEF and right IFSP had a strong positive influence on classification (Figure 3E). The rest of the anatomical regions among the top 20 features were generally from the right insular, cingulate and occipital regions (Figure 3F).

The BNT and AFT were the only two language tests we could model, as there was too great a class imbalance to model AVLT performance. The mean AUC of the BNT was 0.48 ± 0.16 with the full connectome, which was able to rise to 0.82 ± 0.16 with feature reduction. The auditory system was the strongest predictor of performance, followed by the DMN (Figure 3G). A positive correlation between the right STSdp, a DMN parcel, and left 23d, a CEN parcel, along with the right 8Ad and right TPOJ1, both DMN parcels were the top two features contributing to the model’s output (Figure 3H). The rest of the top 20 features were generally right parietal and temporooccipital parcels (Figure 3I).

Finally, the AFT achieved a mean AUC of 0.56 ± 0.10 with the full connectome, and 0.77 ± 0.08 following feature reduction. A number of canonical networks showed high importance in AFT performance, including the DMN, DAN, medial temporal region, VAN, and VN (Figure 3J). There was a large amount of overlap between the features in the SHAP plot, making it difficult to identify a single salient feature, however, the top 20 features included a large number of occipital and insular parcels bilaterally, along with left cingulate regions (Figures 3K,L).

**FIGURE 4:**
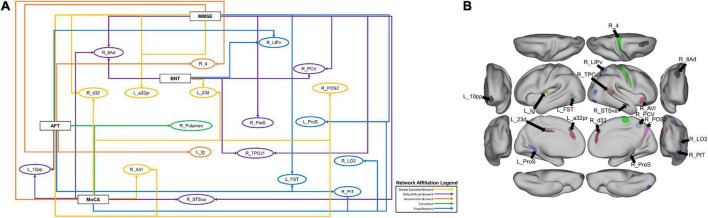
**(A)** A schematic demonstrating the 19 parcels which were within the top 20 features of at least two models classifying neuropsychological test performance. Each arrow goes from a neuropsychological test label to a brain parcel. The color of each arrow indicates the network affiliation of the parcel with which it is associated. The parcels have been placed in rough anatomical space. **(B)** The same parcels, with the exception of the subcortical right Putamen represented on a brain for anatomical reference.

In order to explore further patterns within the top 20 features identified by the machine learning models, we identified parcels which were associated with at least two neuropsychological tests, which comprised 19 individual parcels (Figure 4A). Right area 8Ad, a DMN parcel, and right posterior inferotemporal area (right PIT) were each associated with three tests. Right 8Ad was associated with the BNT, MoCA and MMSE, whereas right PIT was associated with the BNT, AFT and MoCA. At a network level, six of the identified parcels were associated with the DMN, five with the CEN, five with the VN, and two with the SMN. The right putamen was also identified, showing an association with the AFT and MoCA. Anatomically, most of the parcels were in the parietal and occipitotemporal regions Figure 4B).

N.b. because the full sample was included in the analysis, we reran all of the analyses of neuropsychological performance including diagnosis as a covariate, however, these results are not affected by the inclusion of diagnostic category, suggesting that diagnostic category is not accounting for a substantial portion of performance difference, when compared to connectivity differences.

## Discussion

In this study, we investigated whether functional connectivity, coupled with sophisticated machine learning techniques could differentiate SCD, MCI and HC, and reveal key features contributing to the functional deficits. Notably, our models achieved a high performance when differentiating between the diagnostic groups and demonstrated that the language and accessory language networks are impacted early in dementia related pathology, even in the context of normative cognitive function. We also examined the networks and regions underlying cognitive deficits as identified by two general cognitive, and two language tests, demonstrating the recruitment of several central networks, and possibly suggesting nociferous effects of attempted compensation. Finally, a centrality-based examination of our data revealed a hubopathy within deep brain regions, including the basal ganglia and limbic regions, along with the frontal operculum. Together, our findings demonstrate the utility of our methodology in exploring the neural networks underlying functional deficits in pre-dementia status, along with the cognitive and neuroplastic responses to pathology. These findings should be considered exploratory and interpreted with caution as they need to be validated outside of our clinical cohort.

### Using machine learning for dementia diagnosis

Several studies have proposed machine learning approaches for the classification of MCI and AD (Gray et al., 2012; Wee et al., 2012; Nozadi et al., 2018; Billeci et al., 2020; Shi and Liu, 2020; Syaifullah et al., 2020; Sheng et al., 2022). These have utilized either one or combination of neuropsychological test scores, neuroimaging, and biofluids. A large proportion of these studies have however, relied on feature selection in order to extract salient features from a modality prior to the application of machine learning techniques. This in turn simplifies feature importance extraction. In our sample, while feature reduction improved the AUC of some models, presumably by removing potentially redundant parts of the data, it was less reliable for network-based analysis. We therefore based our conclusions on the models utilizing the entire connectome. Nevertheless, the AUC of our models, even when relying on the full set of features, was comparable to previous studies relying on fMRI, which tend to range from 0.75 to 0.90 (Drane et al., 2008; Goryawala et al., 2015; Jie et al., 2018; Nozadi et al., 2018; Zhang et al., 2019; Shi and Liu, 2020). A more complete evaluation of the discriminatory capacity of our techniques would however, need to be conducted using a larger dataset.

### Language networks in pre-dementia status

Our findings demonstrated that functional connectivity of the accessory language network was most associated with models classifying the early stages of cognitive decline from healthy controls. The specific regions that the software labels as accessory language are left area TGv in the temporal pole, and areas STSda, STSva and TE1a in the anterior temporal lobe. These regions are part of the extended DMN and have been associated with semantic memory, which is an impaired function in SCD and MCI.

Evidence of the impact of damage to these regions comes predominantly from temporal lobe epilepsy surgery. Patients undergoing left anterior temporal lobectomy were less efficient in language-dependent tasks, with difficulty in visual naming and verbal memory (Ivnik et al., 1987; Saykin et al., 1995; Drane et al., 2008). This pattern of focal temporal degeneration has also been classically associated with semantic dementia (SD), also known as the temporal variant of frontotemporal dementia (Hodges and Patterson, 2007; Patterson et al., 2007). Patients with SD exhibit onset of gray matter atrophy in the anterior temporal lobes, with predominant left lateralization, which extends with disease progression to the temporal pole, along with the medial and lateral temporal regions (Schwab et al., 2020). This is associated with deficits in semantic memory, with usually intact episodic memory (Bozeat et al., 2000). In contrast, AD has classically been associated with FC changes in the hippocampus and the DMN (Allen et al., 2007; Wang et al., 2007). Although atrophy and cortical thinning has been described in the rest of the temporal lobe in AD, these changes are generally less symmetrized (Galton et al., 2001; Domoto-Reilly et al., 2012; Schwab et al., 2020); though interestingly, it has been demonstrated that TDP-43 pathology, one of the pathological inclusions in AD, appears at a very early stage in the anterior temporal pole in AD (Nag et al., 2018). It is therefore interesting that the accessory language network was highlighted in our dataset. Although it is possible that our subjects may have an overrepresentation of individuals who may convert to semantic dementia, given the relative rarity of this condition, it is more likely that semantic dysfunction is a common deficit in pre-dementia status.

Interestingly, the model specifically classifying SCD from HC highlighted the language network, as opposed to the accessory language network. Previous reports have suggested that pre-dementia status is associated with increased connectivity within the language network (Pistono et al., 2021b), while AD has been demonstrated to have lower FC in the language network compared to controls (Weiler et al., 2014; Montembeault et al., 2019). In healthy aging, the language network also shows an increased FC, and interacts with the CEN and DAN to maintain language performance (Muller et al., 2016; Hoffman and Morcom, 2018; Pistono et al., 2021a). Together, this pattern may be indicating that the prominence of the language networks in our sample is indicative of a protective mechanism differentiating HC from pre-dementia status, where this compensation is lost early in AD. However, further studies specifically examining language networks in the context of AD are necessary. Furthermore, longitudinal studies should examine the conversion of individuals with language network dysfunction to AD and other types of dementia.

In contrast, the networks most associated with the model differentiating SCD and MCI highlighted the DMN and CEN, suggesting that MCI is characterized by extension of network dysfunction to executive networks. This finding is in line with neuropsychological studies examining the temporal course of cognitive decline in AD, suggesting that semantic memory deficits occur earlier than episodic memory decline (Amieva et al., 2008; Howieson et al., 2008), arising as early as 4 years prior to MCI diagnosis in one study (Mistridis et al., 2015). Consequently, techniques such as ours may be able to integrate a structural pathological model of dementia with functional changes along the spectrum of dementia.

### Parcel to neuropsychological test mapping

Mapping parcels to specific neuropsychological tests demonstrated that neurocognitive deficits in pre-dementia status are associated with FC changes in multiple bi-hemispheric neural networks. However, a key consideration in the application of this technique is whether it provides any justifiable utility beyond the use of neuropsychological testing, which is significantly easier to implement in a clinical environment. In our sample, the relationship between raw language test scores and diagnostic groups was only statistically significant between the MCI and HC groups. Our methods may therefore have the potential to identify FC changes prior to the onset of functional deficits. Our findings also provide further insight into the anatomical basis of these deficits. For example, while regions associated with semantic memory contributed to the pre-dementia status model, our model of the BNT highlighted the auditory system as the key network associated with this model. It is important to note that since we did not utilize task-based fMRI, these network changes are only an indication of the differences between poor performers and those with preserved function. They therefore have the potential to demonstrate changes indirectly associated with a given function and may potentially be reflecting maladaptive compensation. The goal of this type of analysis is therefore to identify possible neuroimaging markers of disease progression or targets of treatment, though longitudinal data are required to necessitate this.

In line with these potential neuroplastic changes, our model classifying SCD and HC had a large number of sensory regions within its top features compared to the model classifying MCI and HC. There was also an overrepresentation of the visual system’s contribution to the SCD vs. HC model, along with the common regions contributing to neuropsychological test performance. This may either be highlighting early damage to sensory regions in SCD and MCI, or indicating nociferous compensation by sensory regions following damage to associative networks in later stages of the disease. This is congruent to a recent study exploring FC changes in a small cohort of SCD, MCI and AD subjects, where the authors demonstrated a decreased centrality within the SMN and VN in SCD, and increased centrality in these networks in AD, proposing possible compensation of these networks following damage to the DMN, CEN and DAN (Wang et al., 2019). While further longitudinal studies are required to explore this hypothesis, it is evident that a FC-based approach to functional performance may reveal key insights about the anatomical basis of pathological function.

### Limitations

Our study is limited by a small sample size, and therefore our findings require larger scale prospective studies for validation. Additionally, while we employ effective class imbalance mitigation methodologies, we cannot assume that class imbalances do not bias our results, thus we suggest that future research requires larger and balanced samples. AUC as a metric is somewhat sensitive to class imbalance. Nevertheless, our methodology employs approaches to mitigate this risk, including fivefold cross-validation and early stopping criteria. Furthermore, our analysis focuses mainly on feature importance, and therefore we can assume that our protective measures and significantly higher than chance performance make nuances in AUC bias less relevant to the purpose of our analysis. In addition, future studies should investigate longitudinal connectivity changes in MCI and AD to ascertain pathological and compensatory changes. Therefore, while our study highlighted early changes in dementia in a clinical cohort, future studies should aim to replicate these findings and explore whether these techniques may be used to establish diagnostic trajectories or therapeutic targets.

## Data availability statement

The raw data supporting the conclusions of this article will be made available by the authors, without undue reservation.

## Ethics statement

The studies involving human participants were reviewed and approved by Ethics Committee of Jiangsu Shengze Hospital. The patients/participants provided their written informed consent to participate in this study.

## Author contributions

YS and TW conceived and designed the study. QL, TZ, and HY performed the study and collect materials. NM wrote the code. NM and KO analyzed the results. KO and OT visualized the results. OT, IY, YS, and QL wrote the manuscript. IY, MS, SD, XL, XZ, and TW helped coordinate the study and reviewed the manuscript. All authors contributed to the article and approved the submitted version.
